# Supplementary material for: Clostridium perfringens Associated with Foodborne Infections of Animal Origins: Insights into Prevalence, Antimicrobial Resistance, Toxin Genes Profiles, and Toxinotypes
Source: Biology (Basel). 2022 Apr 1;11(4):551. doi: 10.3390/biology11040551 (PMC9028928; doi:10.3390/biology11040551)
Supplement: Supplementary file 1 [file biology-11-00551-s001.zip › biology-1611869-supplementary.pdf]

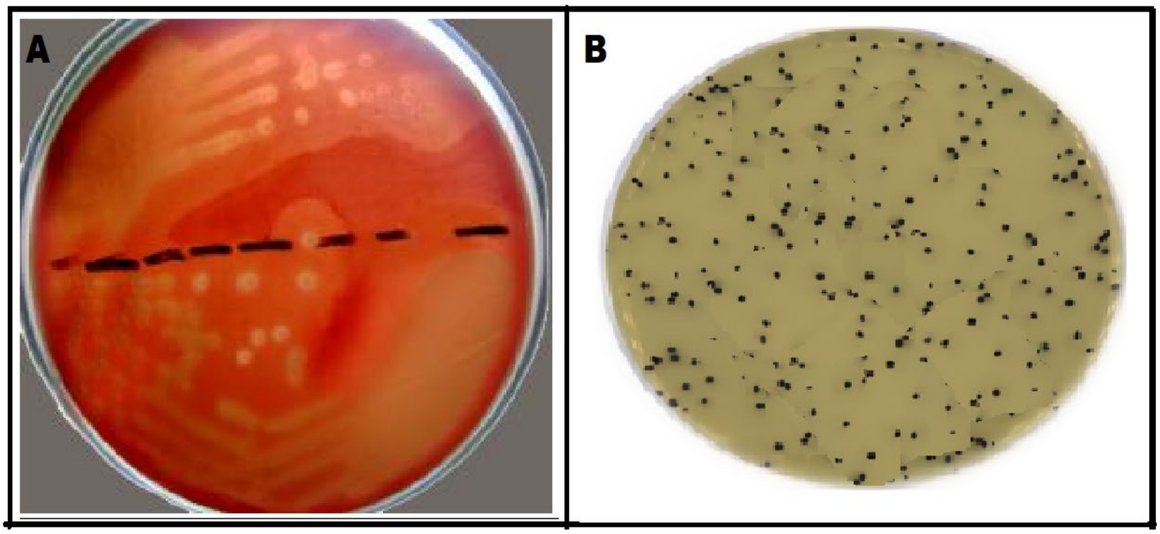

Figure S1. Cultural characters of *C. perfringens* isolates on blood agar (**A**) showing double zone of hemolysis and on tryptose sulfite cycloserine agar (**B**) demonstrating typical characteristic black colonies.

Table S1. Sources, numbers, locality, and distribution of the collected samples in the current study.

Locality	Supermarket Code	Number of Collected Samples			Total
		Raw Milk	Beef	Chicken Meat	
**Sharkia**	A	10	10	10	30
	B	10	10	10	30
	C	10	10	10	30
	D	10	10	10	30
	E	10	10	10	30
	F	10	10	10	30
	G	10	10	10	30
	H	10	10	10	30
**Total**		**80**	**80**	**80**	**240**
**Port Said**	I	10	10	10	30
	II	10	10	10	30
	III	10	10	10	30
	IV	10	10	10	30
	V	10	10	10	30
	VI	10	10	10	30
	VII	10	10	10	30
**Total**		**70**	**70**	**70**	**210**

Table S2. Interpretation of minimum inhibitory concentration values of different antimicrobial agents against *Clostridium perfringens.*

**Antimicrobial Agent**	**Symbol**	**Interpretative Categories and MIC Breakpoints (μg/mL)**	
		**S**	**R**
Ampicillin	AMP	≤4	>8
Amoxicillin/clavulanic acid	AMC	≤4	>8
Cefoxitin	FOX	≤16	≥64
Enrofloxacin	ENR	≤2	≥8
Imipenem	IMP	≤2	>8
Chloramphenicol	C	≤8	>8
Lincomycin	L	≤2	≥8
Metronidazole	MTZ	≤4	>4
Erythromycin	E	≤2	≥8
Tetracycline	TE	≤4	≥16
